# Stathmin in human health and diseases: from cytoskeletal dynamics to clinical implications

**DOI:** 10.3389/fphar.2026.1888339

**Published:** 2026-07-27

**Authors:** Luqi Yang, Lingfei Li, Wan Zhao, Yanhai Feng, Xu Yang

**Affiliations:** 1 Department of Dermatology, Daping Hospital, Army Medical University, Chongqing, China; 2 Department of Dermatology, The 991th Hospital of Joint Logistic Support Force of People’s Liberation Army, Hubei, China; 3 Research Center for Skin Tissue Engineering of Chongqing Higher Education Institutions, Daping Hospital, Army Medical University, Chongqing, China; 4 State Key Laboratory of Trauma and Chemical Poisoning, Daping Hospital, Army Medical University, Chongqing, China; 5 Department of Respiratory and Critical Care Medicine, The Second Affiliated Hospital of Chongqing Medical University, Chongqing, China

**Keywords:** cytoskeleton, dynamics, mechanism, microtubule, pathophysiological role, stathmin

## Abstract

Stathmin, a highly conserved microtubule-destabilizing protein in eukaryotes, plays a pivotal role in regulating cytoskeletal dynamics by promoting microtubule depolymerization. This process is indispensable for fundamental biological events, including cell division, migration, differentiation, and intracellular signal transduction. This review systematically elucidates stathmin’s genomic architecture, molecular functions, and mechanistic contributions under both physiological and pathological contexts. The functional output of stathmin is tightly modulated by post-translational modifications, particularly phosphorylation, as well as its protein expression levels. Aberrant stathmin expression or activity is strongly implicated in tumorigenesis, driving cancer cell proliferation, invasion, metastasis, and chemoresistance. Conversely, in non-neoplastic diseases, stathmin orchestrates skin and mucosal repair, maintains neurological function, influences pulmonary fibrosis, modulates metabolic homeostasis, and supports pregnancy maintenance through its cytoskeletal regulatory capacity. Furthermore, this study highlights stathmin’s significant potential as a diagnostic biomarker and a promising therapeutic target. Finally, we propose future research directions that prioritize deciphering tissue-specific regulatory mechanisms and accelerating the translation of basic scientific discoveries into clinical applications.

## 1 Introduction

Microtubules are fundamental components of the eukaryotic cytoskeleton. Typically composed of 13 protofilaments, these cylindrical polymers exhibit highly dynamic properties, a phenomenon known as dynamic instability. Each protofilament consists of head-to-tail arrays of α-/β-tubulin heterodimers. Microtubules perform essential functions in nearly all eukaryotic cells, including the maintenance of cell morphology, intracellular trafficking, and assembly of the mitotic spindle during cell division.

The stathmin family proteins are ubiquitously expressed across eukaryotes. These proteins share a conserved stathmin-like domain (SLD) characterized by proline- and serine-rich regions. Stathmin promotes microtubule depolymerization through direct binding to α-/β-tubulin heterodimers, thereby regulating critical cellular processes such as proliferation, mitosis, and signal transduction. Dysregulation of stathmin expression or function is frequently associated with various pathologies, including oncogenesis and neurodegenerative disorders.

## 2 Gene and protein structure of stathmin

**FIGURE 1:**
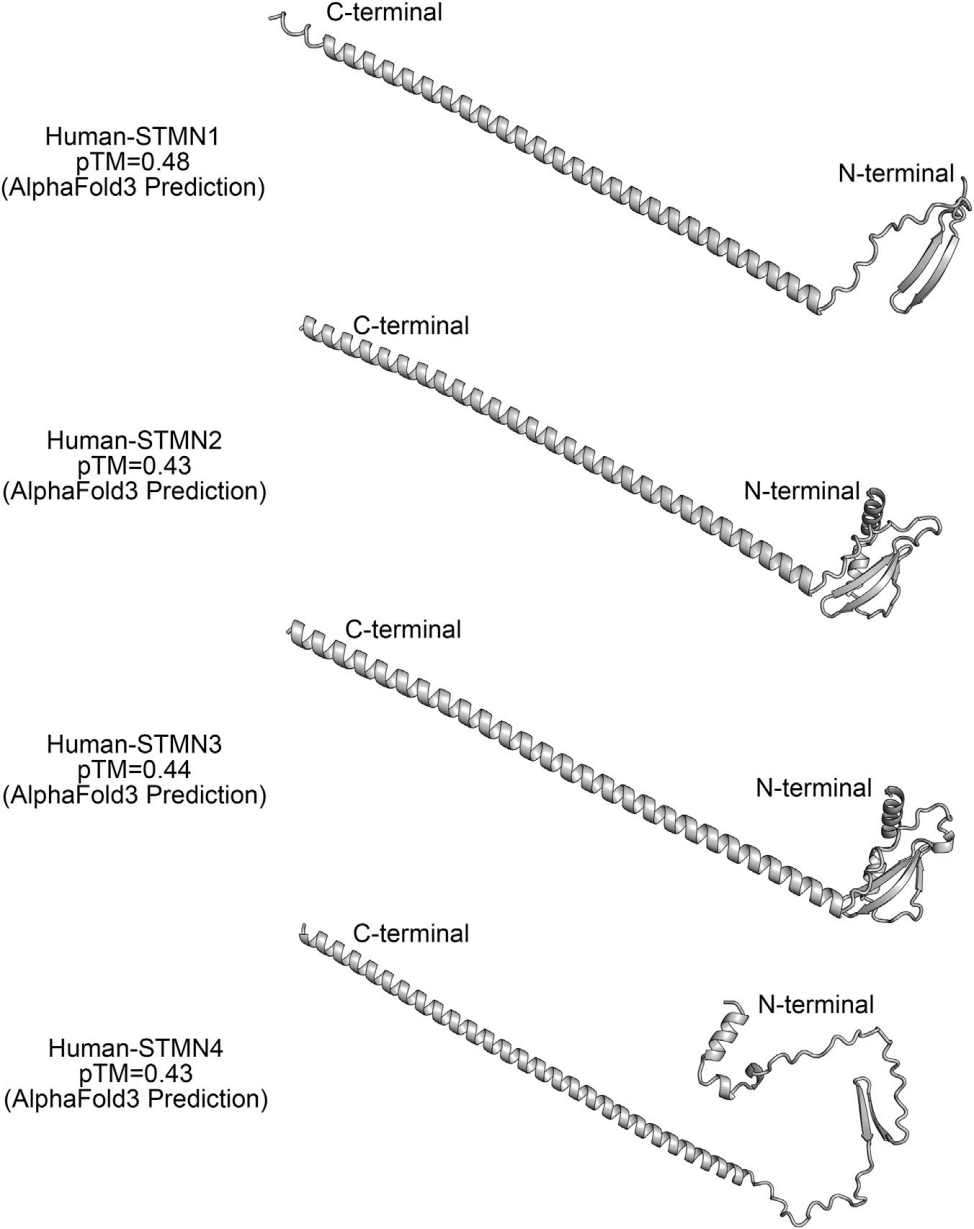
AlphaFold3-Predicted Structures of Human Stathmin (STMN) Family Proteins. The predicted three-dimensional structures of human STMN1, STMN2, STMN3, and STMN4 were generated using AlphaFold3 (with predicted TM-scores: STMN1 = 0.48, STMN2 = 0.43, STMN3 = 0.44, STMN4 = 0.43). The N-terminal (variable domain) and C-terminal (conserved helical domain) regions are labeled. STMN1–3 exhibit a long C-terminal α-helical stalk with a compact N-terminal fold, whereas STMN4 shows a distinctively folded N-terminal domain and a shorter helical C-terminus, highlighting structural divergence within the stathmin family.

**FIGURE 2:**
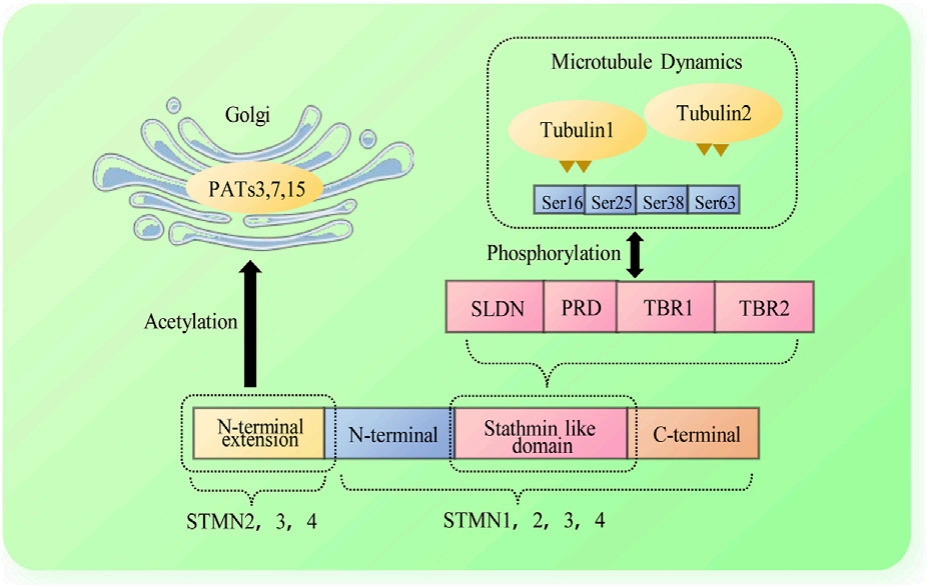
The structure of stathmin and the distinct regions of its N-terminal and C-terminal domains. This schematic illustrates the domain architecture of stathmin family proteins (including STMN1, STMN2, STMN3, and STMN4) and their post-translational modifications (acetylation and phosphorylation) that modulate microtubule dynamics. Stathmin family members are divided into two major structural groups based on their N-terminal regions: STMN2, 3, and four contain an additional N-terminal extension (orange box) preceding the N-terminal domain (blue box), whereas STMN1, 2, 3, and four all possess a conserved stathmin-like domain (pink box), which is responsible for microtubule destabilization, followed by a C-terminal domain (brown box). Post-translational modifications and functional regulation: the N-terminal extension of STMN2/3/4 is subject to acetylation (indicated by the black arrow), which is associated with Golgi localization and involves PATs3, 7, and 15; the stathmin-like domain contains specific functional motifs including SLDN, PRD, TBR1, and TBR2, which undergo phosphorylation (indicated by the bidirectional arrow) at specific sites (Ser16, Ser25, Ser38, and Ser63) on two tubulin heterodimers (tubulin1 and tubulin2), thereby regulating microtubule dynamics.

Stathmin, also known as STMN1, OP18, or SCG10, is encoded by a gene on human chromosome 1p35∼36.1. This gene spans 6.3 kb and contains 4 introns and 5 exons (Zhu et al., 1989); translation starts near the end of the second exon. Stathmin is a small phosphoprotein, with a molecular weight of approximately 19–22 kDa and comprises roughly 163 amino acids (Figure 1). By binding to the β-subunit of tubulin, stathmin restrains microtubule growth and promotes their disassembly, thereby altering microtubule dynamics (Charbaut et al., 2001). All members of the stathmin family (stathmins 1–4) share a conserved region that bind two tubulin dimers, forming a tubulin-stathmin complex. Notably, stathmins 2–4 contain an extra N-terminal domain that helps anchor them to membranes. Within this domain, two cysteine residues (the CC motif) can be modified by specific palmitoyl acyltransferases (PATs 3, 7, and 15) in the Golgi apparatus (Figure 2). The N- and C-terminal portions of stathmin perform distinct functions in molecular interactions (Charbaut et al., 2001) (Table 1).

**TABLE 1:** Comparative overview of the stathmin protein family (STMN1–4).

Feature	STMN1 (Stathmin/Op18)	STMN2 (SCG10)	STMN3 (Stathmin-3)	STMN4 (Stathmin-4)
Tissue specificity	Ubiquitous (high in tumors)	Neuron-specific	Brain, testis, tumors	Ubiquitous (low expression)
Key domains	SLD (stathmin-like domain)	SLD + N-terminal CC motif	SLD + N-terminal CC motif	SLD + N-terminal CC motif
Subcellular localization	Cytosol, nucleus	Golgi apparatus, axons	Golgi apparatus	Golgi apparatus
Regulation	Phosphorylation (Ser16, 25, 38, 63)	Palmitoylation (Cys22/24)	Palmitoylation	Palmitoylation
Knockout phenotype	Anemia, thrombocytosis, renal fibrosis	Axon degeneration, ALS risk	Embryonic lethal (mice)	Mild phenotype
Primary function	Cell cycle, division, migration, et al.	Axon growth, neuronal plasticity, et al.	Development, differentiation, et al.	Cytoskeletal modulation, et al.

This table summarizes the genomic location, tissue and cell-type expression specificity, structural features, subcellular localization, major post-translational regulatory mechanisms, primary molecular functions, knockout phenotypes, and disease associations for the four stathmin family members. STMN1 is the most widely expressed and extensively studied isoform, whereas STMN2 exhibits the strongest neuron- and axon-specific functions. STMN3 is critical during early embryonic development, and STMN4 appears to play a fine-tuning role in cytoskeletal dynamics. The diversity in expression profiles and functional outcomes underscores the need to consider family-wide redundancy and divergence when interpreting stathmin-related phenotypes.

## 3 Distribution and biological functions of stathmin

### 3.1 Distribution of stathmin

Stathmin was first characterized as a ubiquitous intracellular phosphoprotein in the 1980s, initially identified in rat brain and pituitary tissues through its phosphorylation in response to extracellular signals. It localizes predominantly to the cytoplasm and cytoskeleton, where it regulates microtubule dynamics, with additional nuclear pools detected in various cell types, and is widely expressed across vertebrate species (Sobel et al., 1989).

### 3.2 Stathmin and the cytoskeleton

The cytoskeleton is a fibrous protein matrix composed of microfilaments, microtubules, and intermediate filaments (Don et al., 2004). By binding to α/β-tubulin heterodimers, stathmin significantly influences the polymerization and depolymerization of microtubules, thereby regulating the structure and function of the cytoskeleton.

### 3.3 Stathmin in cell cycle, proliferation, and differentiation

**FIGURE 3:**
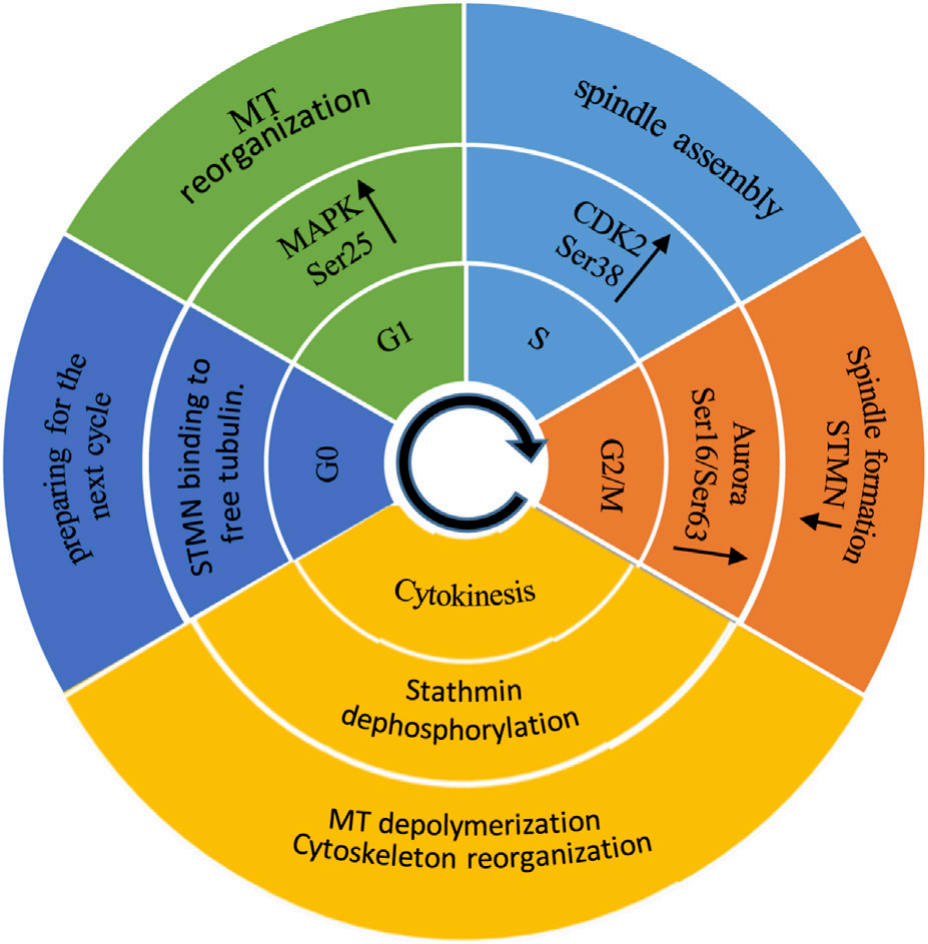
The role of stathmin during the cell cycle. This circular diagram illustrates the dynamic changes in stathmin phosphorylation and its corresponding roles in microtubule (MT) dynamics across different cell cycle phases. During the G1 phase, stathmin is phosphorylated at Ser25 MAPK, contributing to MT reorganization. Entering the S phase, phosphorylation at Ser38 CDK2 facilitates spindle assembly. In the G2/M phase, Aurora kinase phosphorylates stathmin at Ser16 and Ser63, suppressing its binding to free tubulin and promoting spindle formation. Following mitosis and cytokinesis, cells re-enter the G0 phase where stathmin undergoes dephosphorylation mediated by protein phosphatases (PP1, PP2A, and PP2B). This dephosphorylation enhances stathmin’s binding to α/β-tubulin heterodimers, leading to MT depolymerization and cytoskeletal reorganization, thereby preparing the cell for the next cycle. The diagram highlights how the cyclical phosphorylation and dephosphorylation of stathmin serve as a critical switch regulating microtubule stability and cellular architecture throughout the cell cycle.

Stathmin plays a pivotal role in regulating the cell cycle, proliferation, and differentiation. Throughout the cell cycle, stathmin exerts stage-specific functions primarily by modulating microtubule dynamics (Figure 3). During mitosis, microtubules, nucleated by the spindle poles, capture sister chromatids via kinetochores and subsequently pull them toward the opposite poles of the daughter cells (Larsson et al., 1997). During cell cycle, the activity of stathmin is regulated by post-translation modifications, such as phosphorylation. A distinct phosphorylation sites play different role during cell cycle phases: Ser25 is phosphorylated by MAPK (Mitogen-Activated Protein Kinase) during the G1 phase; Ser38 is phosphorylated by CDK2 (Cyclin-Dependent Kinase 2) as cells progress into the S phase; and both Ser16 and Ser63 are phosphorylated by Aurora kinases during the M phase. This sequential phosphorylation inhibits the binding of stathmin to free tubulin heterodimers, thereby facilitating microtubule remodeling and ensuring proper spindle function.

Upon mitotic exit and entry into the G0/G1 phase, stathmin is dephosphorylated by a cohort of phosphatases, including Protein Phosphatase 1 (PP1), Protein Phosphatase 2 A (PP2A), and Protein Phosphatase 2 B (PP2B). This dephosphorylation restores stathmin’s high-affinity binding to α/β-tubulin heterodimers, which promotes microtubule depolymerization and reinstates the dynamic instability of the microtubule network (Ng et al., 2010; Hayashi et al., 2006). Consequently, the dynamic regulation of stathmin phosphorylation is essential for the reorganization of the microtubule network and the concomitant reshaping of the cytoskeleton during cell cycle progression (Deford et al., 2025).

Previous studies showed that the expression of stathmin is low during G1 phase but rises significantly in the S and G2/M phases, the transcription and translation of stathmin can be activated following phytohemagglutinin stimulation, which led to lymphocytes proliferation (Melhem et al., 1991; Hosoya et al., 1996). This pattern suggests that higher stathmin levels is correlated with the most active stages of biosynthesis and division during cell cycle.

During cell differentiation, stathmin expression and phosphorylation status undergo marked alterations (Zheng et al., 2023). Elevated stathmin levels are generally correlated with a proliferative phenotype (Tritschler et al., 2017); conversely, its expression and activity progressively decline as differentiation proceeds. In scenarios requiring active proliferation, increased stathmin expression or activation ensures dynamic microtubule remodeling essential for mitotic spindle assembly (Zhao et al., 2014). In contrast, upon the initiation of differentiation, reduced stathmin expression or activity promotes microtubule polymerization, thereby facilitating the formation of differentiation-specific cytoskeletal architectures. This bidirectional regulatory mechanism positions stathmin as a pivotal molecular switch linking the cell cycle to cellular differentiation. In most physiological differentiation processes, including neurogenesis, myogenesis, and osteogenic differentiation, stathmin expression is downregulated following differentiation onset. Functional inhibition of stathmin typically results in enhanced microtubule network polymerization and subsequent cellular maturation. However, the regulatory role of stathmin during differentiation can be context-dependent, exhibiting distinct patterns under certain physiological or pathological conditions. For instance, during cutaneous wound healing, stathmin facilitates microtubule disassembly to drive the differentiation of dermal fibroblasts into myofibroblasts, thereby accelerating wound contraction, granulation tissue formation, and extracellular matrix remodeling (Tang et al., 2026). Similarly, in the odonto/osteogenic differentiation of human dental pulp stem cells, stathmin positively regulates the Wingless-related integration site (Wnt)/β-catenin signaling pathway, actively driving the differentiation process.

### 3.4 Stathmin in cell migration and invasion

During cell migration, stathmin maintains cytoskeletal dynamics through microtubule depolymerization, thereby facilitating cell movement (Sobel et al., 1989). Upon initiation of migration, upregulated stathmin expression or activity promotes microtubule network rearrangement, driving the formation of lamellipodia and filopodia at the leading edge, which is followed by cell migration. Concurrently, stathmin-mediated microtubule depolymerization enhances directional transport of intracellular vesicles and signaling molecules, establishing a crucial role for cell migration (Xu et al., 2019).

In cellular invasion, stathmin facilitates extracellular matrix penetration by regulating microtubule dynamics to modulate matrix metalloproteinase production and secretion, which play critical roles in regulating cell invasion (He et al., 2016). Notably, stathmin is overexpressed in various malignant tumors, with expression levels positively correlated with tumor cell invasive capacity. This phenomenon may be attributed to stathmin’s dual role in not only promoting cellular motility but also integrating multiple signaling pathways, such as MAPK and Akt (Protein Kinase B) pathways, to synergistically enhance the invasive phenotype.

However, the regulatory effect of stathmin on cell migration exhibits cell-type dependency (Cardama et al., 2017). In different cellular systems, the expression levels, subcellular localization, and interaction patterns of stathmin with microtubules and other cytoskeletal proteins exhibit significant heterogeneity, leading to divergent or even opposing regulatory effects on migratory behavior. For instance, in certain epithelial-derived tumor cells, elevated stathmin expression generally promotes cell migration and invasion by enhancing microtubule dynamics; conversely, in some neuronal or endothelial cells, excessive stathmin activity may disrupt microtubule polarity and thereby impair directional migration. Such functional heterogeneity indicates that stathmin is not simply a “pro-migratory factor”; rather, its biological effects are highly depend on the cellular signaling context, microenvironment, and the status of migration-related pathways. Elucidating the regulatory networks of stathmin across different cell types is therefore essential for understanding its pathophysiological roles.

### 3.5 Stathmin in autophagy and senescence

As a key regulator of microtubule dynamics, stathmin plays a complex and critical role in both cellular autophagy and senescence by affecting cytoskeletal reorganization and signal transduction processes (Wei et al., 2024).

In the regulation of autophagy, the expression level of stathmin is dynamically linked to autophagic activity. Under conditions of cellular stress or nutrient deprivation, higher levels of stathmin phosphorylation help stabilize the microtubule network. This is important for the formation and transport of autophagosomes. Stathmin can also directly help autophagic vesicles mature by interacting with proteins that are involved in autophagy, such as LC3 (Microtubule-associated protein one light chain 3).

During the senescence process, stathmin expression follows a distinctive biphasic pattern. In early-stage senescent cells, excessive stathmin activation can accelerate cellular senescence by disrupting microtubule stability, resulting in mitochondrial dysfunction and impaired proteostasis (Li et al., 2025). In late-stage senescence, however, reduced stathmin expression diminishes cellular adaptability to stress, thereby heightening their vulnerability to apoptosis.

Recent studies have further revealed that stathmin can indirectly regulate the crosstalk between autophagy and senescence by modulating the mTOR (mechanistic Target of Rapamycin) signaling pathway (Murase et al., 2022; Ju et al., 2020). This suggests that stathmin may function as a molecular switch in cellular fate decisions, balancing the delicate relationship between autophagic protection and senescence.

### 3.6 Regulatory mechanisms of stathmin

**FIGURE 4:**
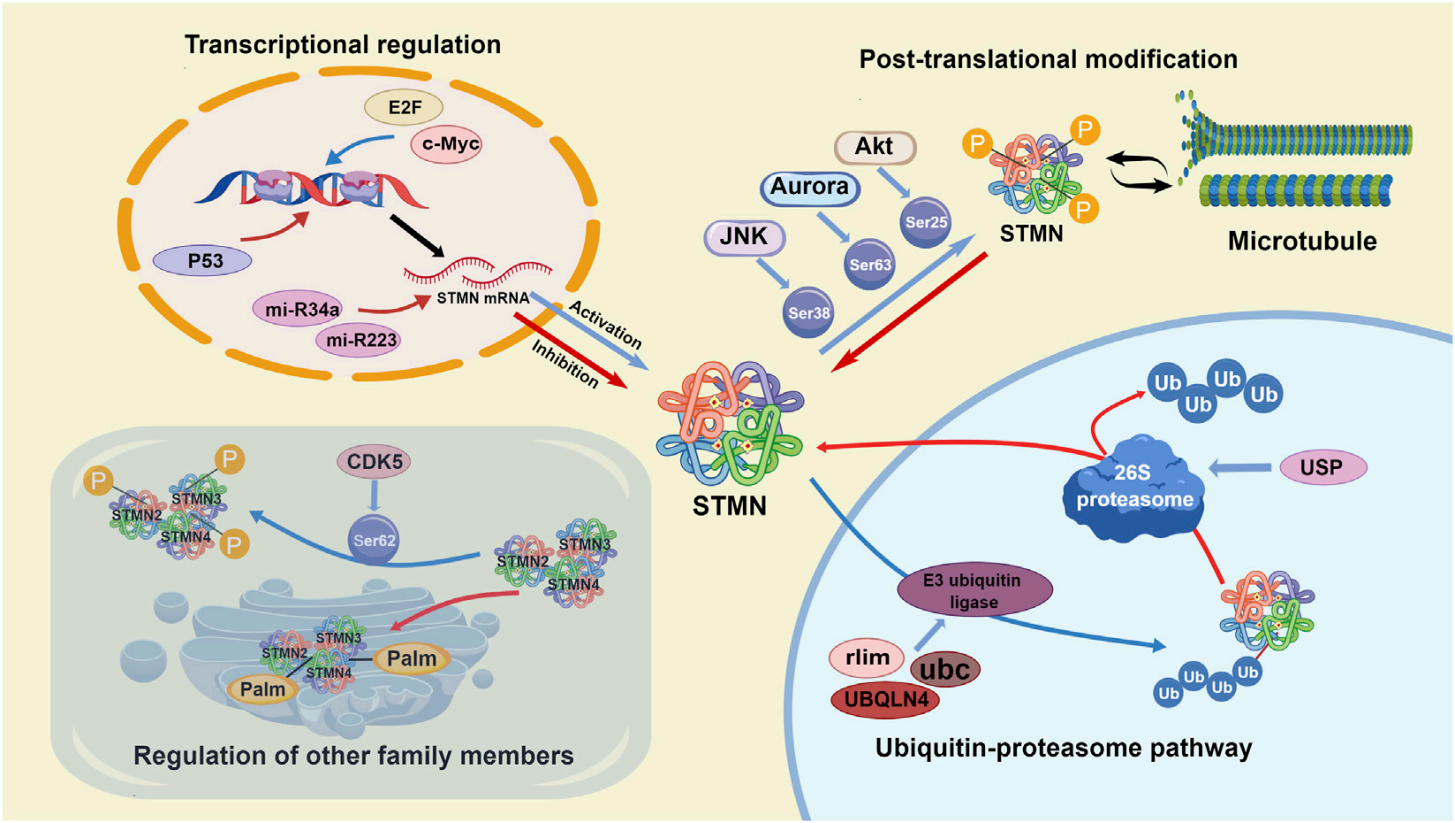
The regulatory mechanisms of stathmin. This diagram illustrates four key layers of regulation: transcriptional control, where transcription factors (E2F, c-Myc, P53) and microRNAs (mi-R34a, mi-R223) modulate STMN mRNA levels; post-translational modification, primarily through the phosphorylation of specific serine residues (Ser25, Ser38, Ser63) by kinases such as MAPK, JNK, Aurora, and Akt, which dictates stathmin’s ability to bind and destabilize microtubules; the ubiquitin-proteasome pathway, involving E3 ubiquitin ligases and deubiquitinating enzymes (like USP) that determine stathmin protein stability and turnover; and the regulation of other family members (STMN2, STMN3, STMN4), which are fine-tuned by factors like CDK5-mediated phosphorylation and palmitoylation for proper subcellular localization and cooperative microtubule regulation.

The biological activity of stathmin is governed by a sophisticated, multi-layered regulatory network that ensures the spatiotemporal precision of its cellular functions. At the molecular level, this regulation includes transcriptional control of STMN1 expression, reversible phosphorylation, proteasomal degradation, and protein-protein interactions. Furthermore, the different members of the stathmin family work in a coordinated manner, collectively regulating their functions (Figure 4).

Transcription factors and microRNAs work together to control the synthesis of stathmin (Figure 4). E2F family transcription factors drive STMN1 transcription; consequently, E2F depletion or inactivation correlates with markedly reduced stathmin expression (Ishida et al., 2001; Polager et al., 2003). Besides, the tumor suppressor p53 stops the expression of stathmin (Johnsen et al., 2000). Additionally, transcription factors like c-Myc increase the expression of stathmin. MicroRNAs including miR-223 and miR-34a target stathmin mRNA, reducing stathmin protein levels in cells either by degrading the mRNA or inhibiting its translation (Masciarelli et al., 2014).

Post-translational modifications play a central role in regulating stathmin function, with phosphorylation representing the most important mechanism (Figure 4). Different protein kinases, like MAPK, Aurora kinases, AMPK, and Akt/PKB, change the way stathmin binds to microtubules and destabilizes them by adding a phosphate group to certain serine residues, such as Ser25, Ser63, and Ser38. For example, phosphorylation of Ser25 mediated by the MAPK/ERK or JNK pathways increases stathmin’s affinity for microtubules (Birnie et al., 2015). In contrast, phosphorylation of Ser63 b y Aurora A/B kinases and phosphorylation of Ser38 b y Akt/PKB or AMPK regulate its microtubule-depolymerizing activity via distinct mechanisms (Lyu et al., 2020). In addition, protein phosphatases like PP1, PP2A, and PP2B can undo these phosphorylation effects by dephosphorylation. This keeps stathmin’s activation state in a dynamic balance. This cycle of phosphorylation and dephosphorylation allows cells quickly respond to signals inside the cell, like energy levels, stress signals, and the progress of the cell cycle. It also allows cells change stathmin’s control over microtubule dynamics in real time, which affects important cellular processes including cytoskeletal reorganization, cell migration, and division.

The ubiquitin-proteasome pathway plays a vital role in regulating stathmin protein homeostasis (Figure 4). E3 ubiquitin ligases identify stathmin and facilitate its ubiquitination, resulting in subsequent degradation by the 26 S proteasome. For instance, rlim overexpression greatly increases stathmin ubiquitination and speeds up its breakdown (Li et al., 2015). Some deubiquitinating enzymes, like USP family proteins, can stabilize stathmin by taking off ubiquitin chains. This makes its functional half-life longer. Ubiquitin C (UBC) and ubiquitin 4 (UBQLN4) have also been linked to stathmin ubiquitination, this pathway keeps a balance between degradation by ubiquitination and stabilization by deubiquitination, carefully adjusting the amount of stathmin protein and making sure that stathmin levels are right for different physiological conditions.

The other members of the stathmin family, STMN2, STMN3, and STMN4, do not work in isolation. They’re part of pretty intricate control networks themselves (Figure 4). Take transcriptional regulation, for example,: a DNA element called the neural restrictive silencer element (NRSE) seems to help tune the expression of STMN2 and STMN3 (Namikawa et al., 2006). For phosphorylation: enzymes like Cyclin-dependent kinase 5 (CDK5) can tag STMN2/3 on a specific site (Ser62), fine-tuning what they do. And for getting to the right place in the cell, proteins like STMN2, STMN3, and STMN4 rely on lipid tags added by palmitoyltransferases at sites called Cyc22/24, this steers them toward the Golgi or the tips of growing processes (Chauvin et al., 2008). All these cooperative controls suggest that stathmin family can adjust microtubule behavior in layered, time- and location-specific ways, and involves in series of diseases.

## 4 Pathophysiological roles of stathmin

### 4.1 Stathmin in skin and mucosa

The skin and mucosal membranes act as the body’s primary external barriers, shielding internal tissues from physical and chemical insults. Stathmin, a microtubule-destabilizing protein, modulates cytoskeletal dynamics to regulate epithelial cell proliferation, differentiation, and migration, which sustain barrier integrity and drive wound healing (Xie et al., 2021). Stathmin expression is tightly regulated under normal conditions and is involved in the normal renewal and differentiation of basal cells (Camoletto et al., 2001). In response to physical or chemical injury of mucosal tissues, stathmin expression is temporarily upregulated, promoting epithelial cell migration and wound closure by enhancing microtubule dynamics (Mohamad Hasan et al., 2025).

**FIGURE 5:**
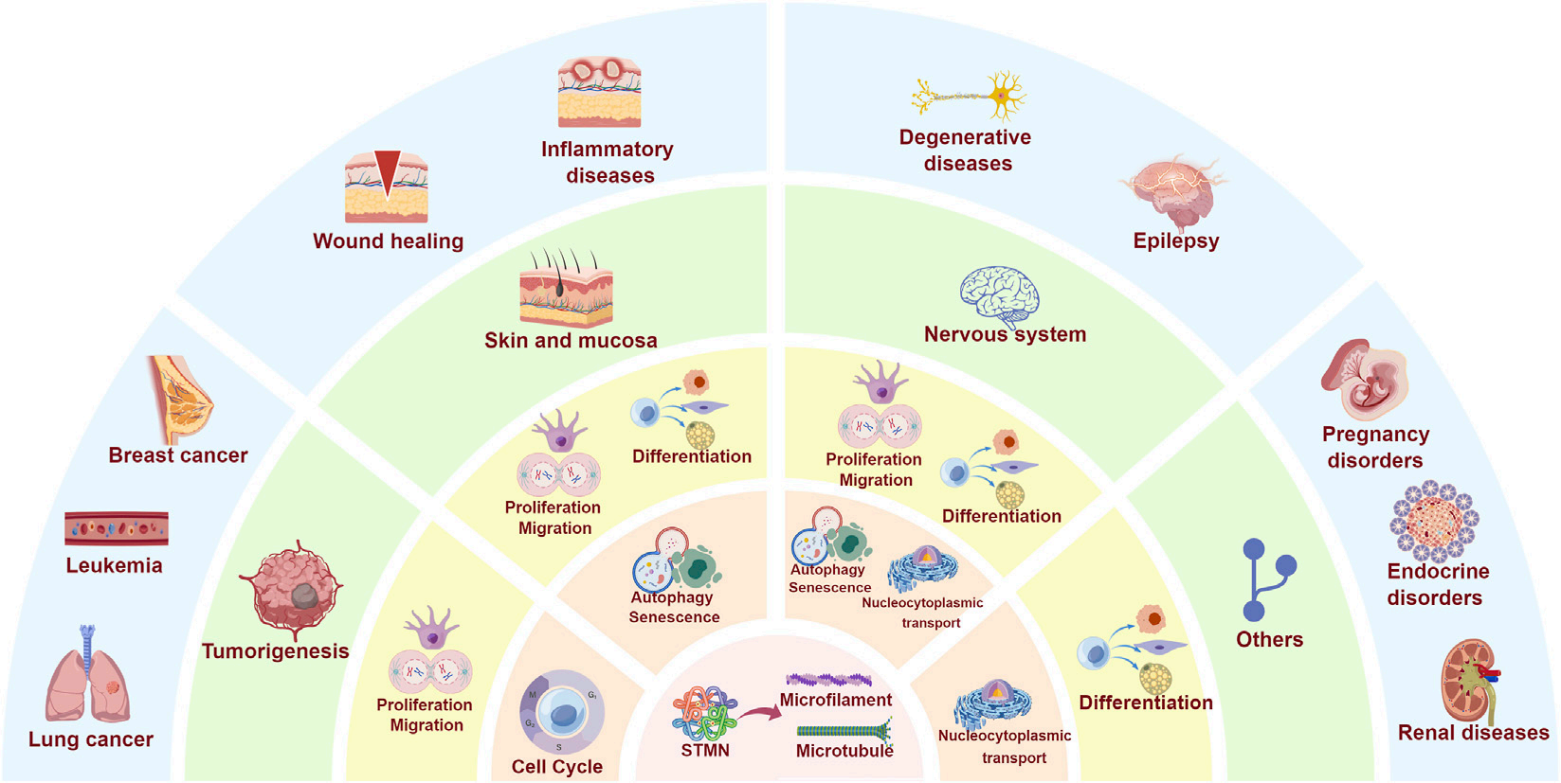
The pathophysiological role of stathmin. This diagram categorizes the involvement of stathmin in conditions such as inflammatory diseases, degenerative disorders, nervous system pathologies (including epilepsy), skin and mucosal integrity, pregnancy-related disorders, endocrine dysfunction, renal diseases, and cancers (breast, lung, leukemia) as well as general tumorigenesis. It further highlights the underlying cellular mechanisms modulated by stathmin, including cell cycle progression, proliferation, migration, differentiation, autophagy, senescence, nucleocytoplasmic transport, and the regulation of cytoskeletal elements (microfilament and microtubule), demonstrating its broad impact on tissue repair, development, and disease pathogenesis.

However, when this regulation system gets out of control, it can lead to skin and mucosa diseases. In conditions like psoriasis, keratinocytes growth rapidly, while stathmin also shows robust increased expression or activity. That overactivity pushes keratinocytes to multiply nonstop and mature in a messed-up way, causing the epidermis to thicken abnormally and weakening the skin’s protective barrier (Schmitt et al., 2013). Aberrant stathmin overexpression is also observed in premalignant lesions such as oral leukoplakia. Persistent elevation of stathmin is tightly correlated with dysplastic and disordered epithelial hyperplasia. By disrupting microtubule dynamic instability, supraphysiological stathmin levels induce cytoskeletal disorganization, thereby promoting genomic instability and driving the malignant transformation of epithelial cells (Ju et al., 2020). It is also noteworthy that stathmin participates in mucosal tissue inflammation, primarily by regulating immune cell motility and activation, which in turn remodels the local inflammatory milieu. In chronic wounds, such as diabetic skin ulcers, stathmin expression is frequently downregulated; this reduction impairs cellular migration and delays tissue repair (Cen et al., 2022). In the context of skin infections, stathmin has been shown to facilitate the recruitment of tubulin to the actin machinery of *Listeria*, which in turn supports the pathogen’s growth (Costa et al., 2019). Together, these insights suggest the central role of stathmin in the integrity maintenance of skin and mucosal barriers (Figure 5), and providing a therapeutic target for specific skin and mucosal diseases.

### 4.2 Stathmin and cancer

Stathmin is a protein that is often not expressed properly in many types of tumors. It is very important for starting, growing, invading, and spreading tumors (Figure 5). High levels of stathmin in cancer cells are often linked to tumors that are more likely to spread and invade other parts of the body (Po’uha et al., 2020). Stathmin affects the reorganization of the cytoskeleton by encouraging the microtubules disassembly. This is important for cancer cells to move and invade other cells. Stathmin also helps control the cell cycle, and changes in its activity, especially during mitosis, lead to uncontrolled growth in tumor cells (Liao et al., 2023).

There are many signaling pathways that control the expression and activity of stathmin. These pathways include kinases and phosphatases that are linked to the cell cycle. In cancer cells, these pathways are frequently dysregulated, resulting in abnormal phosphorylation and functional impairment of stathmin, which subsequently influences tumor cell behavior (Li et al., 2011).

In tumor diagnosis, stathmin expression is significantly elevated in high-grade neuroendocrine tumors (HGNETs) of the lung compared to non-small cell lung cancer (NSCLC), rendering it a useful diagnostic marker for differentiating these tumor types. Similarly, increased stathmin expression serves as a prognostic indicator in breast cancer and strongly correlates with advanced clinical stage, histological grade, and lymph node metastasis in cervical cancer, thereby indicating the risk stratification and clinical prognosis (Brattsand, 2000; Xi et al., 2009). In the hematopoietic system, elevated stathmin expression significantly contributes to the phenotypic transformation of leukemia cells, and its inhibition diminishes the tumorigenic potential of these cells *in vivo* (Jeha et al., 1996).

In terms of cancer treatment and prognosis, high expression of stathmin promotes the proliferation of oral squamous cell carcinoma (OSCC) cells and reduces their sensitivity to 5-fluorouracil (5-FU) chemotherapy (Camoletto et al., 2001). It also enhances resistance to chemotherapeutic agents such as paclitaxel and vinblastine in esophageal squamous cell carcinoma (ESCC) cells (Liu et al., 2013). High levels of phosphorylated stathmin (Ser38) are associated with poor prognosis and correlate with increased tumor cell proliferation, independent of other clinicopathological features (Wik et al., 2013).

### 4.3 Stathmin and the kidneys

The maintenance of renal function relies on the dynamic regulation of the cytoskeletal system. Stathmin directly modulates the cellular morphology and structural integrity essential for glomerular filtration and tubular reabsorption by governing microtubule dynamics (Kumaran and Hanukoglu, 2024).

By orchestrating microtubule assembly and disassembly, stathmin regulates the architecture and physiological function of renal cells, particularly in glomerular podocytes (Figure 5). Podocytes extend specialized foot processes that are critical for the filtration barrier, and stabilized microtubule networks are indispensable for maintaining the structural integrity of these delicate processes (Welsh and Saleem, 2011). Dysregulation of stathmin expression or activity alters podocyte morphology, compromises filtration capacity, and leads to proteinuria. Furthermore, under renal ischemic conditions, aberrant stathmin expression is associated with exacerbated tubular interstitial fibrosis and impaired recovery of tubular structure and function (Zahedi et al., 2006; Zahedi et al., 2004).

### 4.4 Stathmin and pregnancy

In embryonic development, reduced stathmin expression inhibits trophoblast cell proliferation, migration, and differentiation induced by dibutyryl cyclic adenosine monophosphate (db-cAMP) (Yoshie et al., 2008; Tian et al., 2015), leading to neural tube closure, organogenesis, or cell migration (Figure 5).

In maternal tissue adaptation, stathmin expression is tightly regulated to maintain uterine microenvironment homeostasis (Koppel et al., 1999). Dysregulated stathmin expression may disrupt embryonic development by interfering with microtubule dynamics. Studies suggest that stathmin overexpression can impair mitotic spindle formation in early embryos, thereby elevating the risk of aneuploidy, a mechanism potentially linked to unexplained recurrent pregnancy loss (Tamura et al., 2006). During placentation, stathmin modulates trophoblast proliferation and invasiveness. Appropriate stathmin expression supports physiological endometrial invasion, whereas insufficient or excessive expression is associated with preeclampsia or abnormal placental implantation, respectively (Lekva et al., 2021). Moreover, stathmin participates in regulating uterine smooth muscle contractility, its dysregulation may alter microtubule stability in the myometrium, affecting coordinated uterine contractions and potentially playing a role in the pathogenesis of preterm labor or dysfunctional labor (Allen et al., 2015).

### 4.5 Stathmin and the hematopoietic system

In the hematopoietic system, the proliferation, differentiation, migration, and morphologic maintenance of various cell types depend on a precisely regulated microtubule network. As a key modulator of microtubule dynamics, stathmin participates in hematopoietic homeostasis by regulating the proliferation, differentiation, and apoptosis of blood cells. Under physiological conditions, stathmin expression is cell-type specific: it is maintained at relatively high levels in hematopoietic stem and progenitor cells to support self-renewal and rapid proliferation, while its expression gradually decreases during lineage commitment to allow cell-cycle exit and terminal differentiation (Iancu et al., 2000). During erythrocyte maturation, stathmin contributes to cytoskeletal remodeling, which is essential for the morphological transformation of red blood cells into their characteristic biconcave disc shape. In mice, deletion of stathmin leads to reductions in erythrocytes, megakaryocytes, and platelets (Ramlogan-Steel et al., 2021). Additionally, stathmin is involved in the migration and immune responses of leukocytes, particularly lymphocytes, as dynamic microtubule rearrangement is required for cell migration and polarization.

Under pathological conditions, dysregulated stathmin expression emerges as an important feature of hematologic malignancies (Figure 5). In acute leukemia, stathmin overexpression not only promotes aberrant proliferation by destabilizing microtubules but also increases chromosomal instability by disrupting proper mitotic spindle assembly (Machado-Neto et al., 2025). In acute myeloid leukemia (AML), high stathmin levels are significantly associated with chemotherapy resistance, a mechanism potentially linked to stathmin-mediated alterations in microtubule dynamics that affect drug targeting and transport (Xu et al., 2019). In multiple myeloma, stathmin regulates myeloma cell migration and adhesion, facilitating tumor cell infiltration and survival within the bone marrow microenvironment (Wang et al., 2024). Stathmin also participates in megakaryocyte differentiation and platelet production, and its abnormal expression may lead to defects in platelet morphology and function (Iancu-Rubin et al., 2011). Notably, stathmin expression has emerged as a prognostic indicator in certain hematologic malignancies, with high expression often predicting poorer clinical outcomes (Machado-Neto et al., 2014).

### 4.6 Stathmin and the nervous system

Stathmin plays a complicated, two-sided role in the nervous system, on one hand, it is part of normal brain development; on the other hand, it gets tangled up in a range of neurological disorders (Figure 5). Under healthy conditions, stathmin helps control microtubule dynamics, supporting key events like neuronal differentiation, neuron movement, axon pathfinding, and how synapses change over time. Its levels are highest early in development, then taper off as neurons settle into their mature forms (Paolo et al., 1996). As the nervous system wires up, shifts in stathmin activity can sway the growth cone and steer axons, ultimately shaping how neural circuits are built (Beccari et al., 2025). In the adult nervous system, stathmin continues to play a role in neurotransmission, which underlies adaptive changes in neural function and the formation of learning and memory. For example, STAT3 and stathmin can reduce the expression of interleukin-6 (IL-6) through protein–protein interactions, subsequently slowing anterograde axonal transport and altering microtubule stability (Wareham et al., 2021).

Under pathological conditions, aberrant expression of stathmin becomes a significant driver of neurological diseases. In Alzheimer’s disease, stathmin overexpression disrupts microtubule stability, exacerbates abnormal phosphorylation of the microtubule-associated protein tau, and promotes the formation of neurofibrillary tangles, while also accelerating cognitive decline by impairing synaptic vesicle transport (Agra Almeida Quadros et al., 2024; Melamed et al., 2019). In epileptic models, stathmin-mediated hyper-dynamicity of microtubules alters neuronal excitability and lowers the seizure threshold, whereas inhibiting stathmin expression significantly reduces epileptiform discharges (Zhang et al., 2015). Notably, in gliomas, stathmin expression correlates positively with tumor malignancy; it not only enhances tumor cell invasion and migration by promoting microtubule depolymerization but also accelerates glioma cell proliferation by modulating cell cycle (Johnsen et al., 2000). In diseases such as amyotrophic lateral sclerosis (ALS), loss or dysfunction of stathmin constitutes a central mechanism underlying axonal degeneration and motor impairment (Thornburg-Suresh and Summers, 2024).

### 4.7 Stathmin and pulmonary diseases

The structural and functional integrity of the lung relies on tightly regulated cytoskeletal dynamics within alveolar epithelial cells, fibroblasts, and pulmonary vascular endothelial and smooth muscle cells. Stathmin modulates these processes predominantly through the promotion of microtubule depolymerization, which is essential for cellular remodeling during injury and repair.

In pulmonary fibrotic disease, stathmin is upregulated by profibrotic mediators such as TGF-β1. Mechanistically, TGF-β/Smad2/3 signaling increases stathmin expression, and the resultant microtubule destabilization facilitates the cytoskeletal reorganization required for epithelial–mesenchymal transition (EMT) of alveolar epithelial cells and the activation of resident fibroblasts into α-SMA-positive myofibroblasts (Camoletto et al., 2001). By promoting microtubule dynamic instability, stathmin enables the loss of epithelial junctional complexes and acquisition of a migratory, secretory phenotype that drives excessive extracellular matrix deposition characteristic of idiopathic pulmonary fibrosis (IPF) and chronic obstructive pulmonary disease (COPD)-associated fibrosis.

With respect to pulmonary vascular remodeling and pulmonary hypertension (PH), stathmin contributes to the phenotypic switch of pulmonary artery smooth muscle cells (PASMCs) from a contractile to a synthetic, proliferative state via the RhoA/ROCK signaling pathway (Tian et al., 2012). Stathmin-mediated microtubule catastrophe relieves microtubule-associated inhibition of RhoA activation, thereby enhancing ROCK activity, PASMC proliferation, and neointimal formation, which are key features of hypertensive pulmonary vasculopathy. Recent studies further highlight the importance of cytoskeletal regulators in PH pathogenesis; for example, Mas-related G-protein-coupled receptor MrgD negatively regulates PASMC proliferation through the AKT–MAZ–PIM1 axis, suggesting that modulation of cytoskeletal and upstream signaling networks, including stathmin, may influence vascular remodeling in PH (Zhong et al., 2025). Additionally, hyperactivated stathmin exacerbates endothelial barrier dysfunction by amplifying thrombin-induced disruption of adherent junctions and promoting actomyosin contractility, thus increasing vascular permeability (Tian et al., 2012).

In lung cancer, stathmin is aberrantly overexpressed in alveolar epithelial cells and cancer-associated fibroblasts. Elevated stathmin drives malignant progression by promoting microtubule depolymerization that facilitates cell motility, invasion, and EMT​ (Lu et al., 2014). In addition, stathmin also regulate cell cycle progression and apoptotic resistance through the PI3K/Akt signaling axis (Zeng et al., 2024). Clinically, high stathmin expression correlates with advanced stage, increased metastatic potential, and poor overall survival in patients with non-small cell lung cancer (NSCLC) (Lin et al., 2017), supporting its utility as both a prognostic biomarker and a candidate therapeutic target in thoracic oncology.

### 4.8 Stathmin and endocrine diseases

Stathmin family proteins, particularly stathmin-2 (STMN2/SCG10), have emerged as critical regulators of metabolic homeostasis and the pathophysiology of diabetes mellitus (DM). Accumulating evidence indicates that stathmin modulates insulin signaling and glucose transporter trafficking primarily through the PI3K/Akt signaling pathway, thereby influencing both pancreatic β-cell function and peripheral insulin sensitivity.

In pancreatic β-cells, stathmin expression is dynamically regulated during glucose-stimulated insulin secretion (GSIS). Under hyperglycemic conditions, excessive stathmin/STMN2 expression interferes with the PI3K/Akt signaling cascade, leading to impaired Akt (Ser473) phosphorylation and subsequent reductions in insulin synthesis and exocytosis (Demir et al., 2024). Mechanistically, stathmin-mediated microtubule destabilization disrupts the polarized microtubule network required for the proper docking and fusion of insulin granules at the plasma membrane. This impairment compromises GSIS, a process physiologically dependent on glucose-induced microtubule disassembly regulated by Tau phosphorylation (Moser et al., 2005). Studies in rodent models of type 2 diabetes have demonstrated that β-cell-specific downregulation of stathmin restores Akt activation and improves glycemic control, suggesting its negative regulatory role in β-cell compensation (Demir et al., 2024).

Beyond β-cell intrinsic effects, STMN2​ is closely linked to peripheral insulin resistance. In adipose and skeletal muscle tissues, STMN2 levels positively correlate with indices of insulin resistance (e.g., HOMA-IR) and circulating high-sensitivity CRP (Asadi and Dhanvantari, 2021). It has been proposed that STMN2 interacts with the microtubule cytoskeleton to modulate the GLUT4 (glucose transporter type 4) translocation machinery. By altering microtubule dynamic instability, elevated STMN2 delays or impairs insulin-stimulated GLUT4 trafficking to the plasma membrane, a process normally facilitated by intact cortical actin and microtubule networks downstream of Akt activation (Knudsen et al., 2023). Disruption of this microtubule-mediated GLUT4 trafficking is a hallmark of insulin-resistant skeletal muscle (Knudsen et al., 2023).

Clinically, recent studies have reported significantly elevated serum STMN2 levels in newly diagnosed type 2 diabetic patients compared with normoglycemic controls, with positive correlations to fasting insulin, HOMA-IR, and HbA1c (Demir et al., 2024; Asadi and Dhanvantari, 2021). Large-scale transcriptomic analyses further identified STMN2 upregulation in subcutaneous adipose tissue from obese individuals with insulin resistance, linking stathmin family proteins to systemic metabolic dysregulation (Tews et al., 2014). Although the precise regulatory loops governing stathmin in endocrine tissues require further elucidation, current data support its role as both a metabolic regulator and a potential biomarker for insulin resistance.

## 5 Pharmacological targeting of stathmin

Although stathmin (STMN1) is an attractive oncologic and fibrotic therapeutic target due to its central role in microtubule dynamics and signaling crosstalk (e.g., PI3K/Akt, mTOR), direct pharmacological inhibition has historically been challenging given the absence of a classical ligand-binding pocket. Nevertheless, several targeted strategies have emerged:

1. Dual survivin–stathmin (Op18) inhibitor GDP366. GDP366 binds both survivin and stathmin/Op18, destabilizing microtubule networks and inducing G2/M arrest and apoptosis preferentially in transformed cells. In preclinical models, GDP366 exhibited potent anti-proliferative effects in several solid tumors, correlating with stathmin-dependent microtubule disruption (Shi et al., 2010).
2. Chlorambucil–PIP–STMN1 conjugate (Chb–STMN1–PIP). This bifunctional agent couples the alkylator chlorambucil to a pyrrole-imidazole polyamide (PIP) that specifically recognizes the STMN1 gene promoter, thereby repressing STMN1 transcription and delivering localized DNA damage. In small-cell lung carcinoma (SCLC) models, Chb–STMN1–PIP suppressed STMN1 expression, restored microtubule stability, and inhibited tumor growth, illustrating a precision-medicine approach for STMN1-overexpressing malignancies (Yoshikawa et al., 2026).
3. RNA interference–based knockdown. Lipid-formulated short hairpin RNA targeting STMN1 (pbi-shRNA-STMN1 lipoplex) has reached early clinical investigation. By silencing STMN1, this formulation reduces microtubule catastrophizing, dampens cell-cycle progression, and sensitizes tumors to taxane-based chemotherapy. Early phase data support favorable tolerability and proof-of-concept antitumor activity, though optimization of delivery and off-target effects remains ongoing (Wang et al., 2017).
4. Stathmin-derived interfering peptides. Beyond oncology, stathmin-derived peptides exhibit notable antimicrobial potential through the targeting of prokaryotic cytoskeletal proteins. For instance, the peptide I19L, derived from the N-terminal domain of stathmin, disrupts the bundling assembly of FtsZ, a bacterial tubulin homolog essential for cytokinesis via binding proximal to its GTP-binding site. Structural analyses employing NMR spectroscopy and molecular modeling have elucidated distinct interaction modes between I19 L and FtsZ compared to tubulin, thereby highlighting the feasibility of developing selective anti-infective agents based on stathmin mimetics (Clément et al., 2009).

Effective stathmin-targeted therapy holds promise, but still faces challenges: complete abrogation of stathmin activity may cause excessive microtubule stabilization and neurotoxicity, whereas partial modulation may be optimal, particularly in combination regimens (e.g., with PI3K/Akt/mTOR inhibitors or microtubule-stabilizing agents). Biomarker-guided patient selection (e.g., tumors with high STMN1/STMN2 expression) will be essential to maximize therapeutic index. Although no stathmin-only small-molecule inhibitor has yet reached late-stage clinical development, the above strategies underscore its translational promise, especially in STMN1-related cancers and diseases driven by cytoskeletal dysregulation.

## 6 Discussion

In recent years, investigations into stathmin have transcended its initial characterization as a mere microtubule regulator. It is now recognized as a multifunctional protein involved in cell fate determination and the pathogenesis of a diverse array of diseases. Notably, stathmin has emerged as a promising diagnostic biomarker and therapeutic target in various malignancies. Beyond oncology, stathmin modulates neuronal rewiring, cardiovascular physiology, fibrotic progression, and wound healing, underscoring its pervasive role in both homeostatic and pathological processes.

However, the comprehensive regulatory mechanisms and functional networks governing stathmin remain incompletely understood. Key knowledge gaps include its precise role in shaping the immune landscape of the tumor microenvironment. Furthermore, the four stathmin family members (STMN1–4) exhibit distinct tissue-specific expression patterns and divergent biological functions, which have yet to be systematically mapped. From a translational perspective, the development of selective pharmacological modulators that specifically target stathmin isoforms without eliciting off-target effects remains a significant challenge requiring further investigation.
